# Assessment of COVID-19-related olfactory dysfunction and its association with psychological, neuropsychiatric, and cognitive symptoms

**DOI:** 10.3389/fnins.2023.1165329

**Published:** 2023-08-04

**Authors:** Lavandan Jegatheeswaran, Shyam Ajay Gokani, Louis Luke, Gabija Klyvyte, Andreas Espehana, Elizabeth Mairenn Garden, Alessia Tarantino, Basil Al Omari, Carl Martin Philpott

**Affiliations:** ^1^Department of Ear, Nose and Throat Surgery, James Paget University Hospitals NHS Foundation Trust, Great Yarmouth, United Kingdom; ^2^Rhinology and ENT Research Group, Norwich Medical School, University of East Anglia, Norwich, United Kingdom

**Keywords:** olfactory disorders, olfaction, anosmia, COVID-19, hyposmia

## Abstract

**Purpose of review:**

To provide a detailed overview of the assessment of COVID-19-related olfactory dysfunction and its association with psychological, neuropsychiatric, and cognitive symptoms.

**Recent findings:**

COVID-19-related olfactory dysfunction can have a detrimental impact to the quality of life of patients. Prior to the COVID-19 pandemic, olfactory and taste disorders were a common but under-rated, under-researched and under-treated sensory loss. The pandemic has exacerbated the current unmet need for accessing good healthcare for patients living with olfactory disorders and other symptoms secondary to COVID-19. This review thus explores the associations that COVID-19 has with psychological, neuropsychiatric, and cognitive symptoms, and provide a framework and rationale for the assessment of patients presenting with COVID-19 olfactory dysfunction.

**Summary:**

Acute COVID-19 infection and long COVID is not solely a disease of the respiratory and vascular systems. These two conditions have strong associations with psychological, neuropsychiatric, and cognitive symptoms. A systematic approach with history taking and examination particularly with nasal endoscopy can determine the impact that this has on the patient. Specific olfactory disorder questionnaires can demonstrate the impact on quality of life, while psychophysical testing can objectively assess and monitor olfaction over time. The role of cross-sectional imaging is not yet described for COVID-19-related olfactory dysfunction. Management options are limited to conservative adjunctive measures, with some medical therapies described.

## Introduction

Coronavirus disease 2019 (COVID-19), a highly contagious viral illness caused by SARS-CoV-2, resulted in a global pandemic and more than 6.8 million deaths worldwide to date (WHO, 2023). SARS-CoV-2 is an enveloped positive single stranded RNA (+ssRNA) virus, which primarily is transmitted via exposure to respiratory droplets carrying the infectious virus from close contact, or from droplet transmission from pre-symptomatic, asymptomatic or symptomatic individuals incubating the virus (Cascella et al., 2022). Whilst COVID-19 is predominantly considered a respiratory and vascular illness, emerging reports early in the pandemic identified the presence of sudden olfactory loss (anosmia or hyposmia) as being prevalent in patients with COVID-19 (Menni et al., 2020; Parma et al., 2020; Vetter et al., 2020; Gerkin et al., 2021). Despite there being a long association between olfactory and taste disorders during and after viral upper respiratory tract infections including influenza, parainfluenza, rhinoviruses and endemic coronaviruses (Suzuki et al., 2007), it is estimated that the prevalence of anosmia and dysgeusia is 10.2 fold higher and 8.6 fold higher, respectively, in COVID-19 patients when compared to other viral upper respiratory tract infections (Mutiawati et al., 2021). Furthermore, to date there has been over 755 million cumulative COVID-19 cases worldwide, with millions of patients now living with new onset olfactory and taste disorders (Parma et al., 2020; Cecchetto et al., 2021; Mutiawati et al., 2021; Ohla et al., 2022; WHO, 2023).

In addition to the acute symptoms of COVID-19, there are individuals that have the prevalence of these symptoms lasting longer than 12 weeks – this syndrome being termed “long COVID.” Data from the United Kingdom (UK) Census 2021, run by the Office for National Statistics (ONS), places the prevalence of long COVID within the UK population as being between 3.0 to 11.7% (Gokani et al., 2022). Groups at higher risk of developing long COVID include women, those aged 35–49 years old, Caucasian ethnicity or those living with disabilities (Ayoubkhani et al., 2021; Gokani et al., 2022; Ohla et al., 2022).

Prior to the emergence of COVID-19, olfactory and gustatory disorders were a common but under-researched, under-treated and under-rated sensory loss but increasing evidence has shown that anosmia (complete loss of smell) is as an independent risk factor for reduced longevity in this patient cohort (Pinto et al., 2014; Laudisio et al., 2019; Liu et al., 2019). This disease brings with it novel challenges and also highlights and exacerbates the current unmet need for accessing good healthcare for these patients living with olfactory disorders and other symptoms secondary to COVID-19 (Ball et al., 2021). This article thus explores the associations that COVID-19 has on psychological, neuropsychiatric, and cognitive symptoms, and provide a framework and rationale for the assessment of patients presenting with COVID-19 olfactory dysfunction.

## COVID-19 associations

### Cognitive symptoms

Coronaviruses, the broad family of viruses that the SARS-CoV-2 virus belongs to, is one of many pathogens known to cause post-infectious olfactory dysfunction (Suzuki et al., 2007). Nasal epithelial cells show relatively high expression of the angiotensin-converting enzyme 2 (ACE-2) receptor, which is required for the entry of SARS-CoV-2 (Sungnak et al., 2020; Song et al., 2021). SARS-CoV-2 thus can enter the Central Nervous System (CNS) through the olfactory nerve which is the only cranial nerve in contact with the environment. Viral damage of the olfactory bulb may be the first insult needed for further degeneration to occur (Song et al., 2021; Kay, 2022). In the acute phase of COVID-19 infection, autopsy studies have identified the prevalence of neuroinflammation, activation of microglia, neuronal death, and meningeal hyperaemia in post-mortem cortex tissues of COVID-19 patients (Boroujeni et al., 2021; Colombo et al., 2021).

Other hypotheses in literature have been proposed for SARS-CoV-2 route of entry into the CNS. A proposed haematogenous route, which is adopted by coronaviruses and other viruses, include the infection of leucocytes by the virus which allows it to be transported across the blood brain barrier (Koyuncu et al., 2013; Nagu et al., 2021). Consequently, neuroinflammation occurs by the triggered release of proinflammatory chemokines and cytokines resulting in increased blood brain barrier permeability and the easier facilitation of SARS-CoV-2 entry into the CNS (Koyuncu et al., 2013; Nagu et al., 2021). An enteric route has also been proposed, whereby SARS-CoV-2 entry into the CNS occurs as a result of there being a direct connection of the enteric nervous system with the brain via the vagus nerve (Gao et al., 2020). All the routes proposed involve the binding of the SARS-CoV-2 spike protein with the ACE-2 receptor on target cells thus facilitating the entry of the virus into the CNS (Gao et al., 2020; Sungnak et al., 2020; Nagu et al., 2021; Song et al., 2021).

Moreover, studies have reported long term CNS involvement and the prevalence of cognitive impairment in long COVID infection ranging from 25 to 50% (Miners et al., 2020; Miskowiak et al., 2021; Rahman et al., 2021; Chen et al., 2022). One proposed theory for the persistent cognitive impairment seen in long COVID may be secondary to the presence of viral RNA in the brain of long COVID patients and persistent systemic inflammation (Stein et al., 2022; de Paula et al., 2023). Furthermore, structural brain changes found in long COVID anosmic patients include lower concentration of grey matter in the amygdala, insular cortex, parahippocampus, frontal orbital gyrus, olfactory cortex, caudate and putamen (Miners et al., 2020; Campabadal et al., 2023). Other structural changes seen include medial temporal lobe dysfunction involving the hippocampus (Llana et al., 2022), entorhinal and perirhinal cortex (Douaud et al., 2022). The medial temporal lobe is important in multiple cognitive processes including semantic memory and processing of emotions and is also one of the first regions to atrophy in Alzheimer’s disease (AD). Semantic memory is long-term memory and relies heavily on the temporal lobe and structures such as the hippocampus and parahippocampus. At least 20% of patients with COVID-19-related olfactory dysfunction had impaired semantic memory (Gokani et al., 2022). Thus, semantic memory impairment seen in long COVID patients is similar in presentation to patients diagnosed with AD (Fiorentino et al., 2022). Other studies have reported additional impairments of executive functions, attention, memory, information processing and fatigue after acute COVID-19 infection (Braga-Paz et al., 2022; Fiorentino et al., 2022; de Paula et al., 2023). It appeared that these symptoms persisted and were more common after follow up at 4 months.

One study comparing patients with chronic fatigue syndrome with patients with long COVID brain fog showed similarity in cognitive patterns between the two groups (Azcue et al., 2022). Although the underlying neuronal substrate is unknown for chronic fatigue, hypothalamic changes which has been observed in chronic fatigue syndrome and myalgic encephalitis may be responsible for long COVID brain fog (Carruthers et al., 2011). Thus, this implies that SARS-CoV-2 is neuroinvasive and may remain in the brain tissue causing neuroinflammation which increases cognitive burden (Stein et al., 2022; de Paula et al., 2023).

COVID-19-related olfactory dysfunction can be a marker of impending cognitive dysfunction. Further large high quality cohort studies investigating the genetic and biomarkers between cognitive dysfunction, anosmia and severity of acute COVID-19 infection are needed. Future studies should also focus on prevention and identifying at risk patients of cognitive dysfunction within this cohort.

### Neuropsychiatric symptoms

Olfactory dysfunction is a known early sign of many neuropsychiatric disorders, particularly AD and Parkinson’s Disease (Yoo et al., 2018; Rebholz et al., 2020; Cristillo et al., 2021; Azcue et al., 2022). It is hypothesized that the neurodegenerative patterns seen in these disorders begin in the olfactory bulb, which is susceptible to damage from inflammatory processes triggered by viral neuroinvasion (Attems et al., 2014; de Erausquin et al., 2021). Furthermore, COVID-19-related olfactory dysfunction, and the observed pattern of degeneration in the olfactory bulb and limbic brain regions, is similar to that seen in the early stages of AD and Lewy body disease (Kay, 2022). Notably olfactory loss, and many neuropsychiatric disorders are associated with high levels of interleukin-6 (IL-6), an inflammatory marker which is also implicated in the cytokine storm in COVID-19 patients (Gialluisi et al., 2020; de Erausquin et al., 2021). In addition to increased IL-6 levels, an increase in levels of C-Reactive Protein (CRP), IL-1β, IL-2 and Tumour Necrosis Factor (TNF) has been observed in both acute COVID-19 patients and Parkinson’s patients, which may imply that high levels of these markers (as seen in the COVID-19 cytokine storm) are associated with a higher clinical severity risk of Parkinsonian symptoms in acute COVID-19 patients (Qin et al., 2016; Qiu et al., 2019; Gialluisi et al., 2020; de Erausquin et al., 2021). This inflammatory process may have the potential to induce neurological damage such as encephalitis (Gialluisi et al., 2020). The use of IL-1 and IL-6 receptor antagonists such as tociluzimab has been seen to reduce the severity of acute COVID-19 illness in patients, which in turn may reduce the neurological damage that occurs secondary to the cytokine storm (Ghofrani Nezhad et al., 2023).

Moreover, acute COVID-19 and neuropsychiatric disorders share common risk factors such as APOE4 allele homo/heterogeneity, increased age, sex, hypertension, diabetes mellitus and obesity (Ortiz et al., 2022). Apolipoprotein 4 (APOE4) allele homogeneity or heterogeneity may lead to potential cerebrovascular dysfunction and neuroinflammation blood brain barrier leakiness (Zhang and Xie, 2020; Ortiz et al., 2022). Furthermore, the SARS-CoV-2 N protein has been shown to inhibit RIG-1-like pathway. RIG-1 (retinoid acid-inducible gene-1) has been found to have associations with schizophrenia suggesting that coronavirus infection could lead to exacerbation of previous neuropsychiatric illness (Rhoades et al., 2022). Moving forward, more research is required to clarify the exact mechanisms underlying the associations between COVID-19-related olfactory dysfunction and neuropsychiatric disorders.

### Psychological symptoms

Psychological impacts are associated with both acute COVID-19 infection and long COVID. In the acute setting, acute COVID-19 infection has been associated with negative feelings and behaviors such as anxiety, stress, anger, avoidance, and isolation (DeJong et al., 2020). In a cohort study of 461 patients hospitalised with acute COVID-19 infection, Kim et al. (2021) identified the presence of symptoms such as anxiety (16.3%), depression (26.5%), insomnia (33.4%), and suicidal ideation (11.7%). These symptoms significantly improved in the week following hospitalisation (Kim et al., 2021). A fluorodeoxyglucose positron emission tomography (FDG-PET) study on acute COVID-19 patients suggests the increased presence of these psychological symptoms being due to COVID-19-related dysfunction of the cingulate cortex, an anatomical area involved in the processing of emotions, decision making, memory and depression (Hugon, 2022). Studies in literature have also observed high levels of various cytokines that are raised in patients infected with SARS-CoV-2, such as IL-6, TNF-α, IL-1β and ferritin in patients with psychiatric disorders such as depression, post-traumatic stress disorder (PTSD) and obsessive–compulsive disorder (OCD) (Ma et al., 2010; Parker et al., 2015; Lindqvist et al., 2017; Karagüzel et al., 2019). Furthermore, pathological analysis of autopsy specimens of patients with acute COVID-19 infection has identified that Neurolipin-1 (NRP-1) is expressed in olfactory epithelial cells and can potentiate SARS-CoV-2 infectivity and provide a route for CNS penetration of the virus (Cantuti-Castelvetri et al., 2020).

Long COVID also has significant psychological associations. In the 3 months following acute COVID-19 infection, patients are at increased risk of mood and anxiety disorders. Taquet et al. (2021) identified that 5.8% of patients had a new psychiatric diagnosis in the 14–90 days after COVID-19 infection in a retrospective study of 62 354 COVID-19 cases in the USA (Taquet et al., 2021). However, this relationship is complex, with patients with pre-existing psychiatric disorders also at a potential increased risk of long COVID (Kataoka et al., 2023). Studies have also suggested that the psychological impact of acute and long COVID is associated with the severity of the initial acute COVID-19 infection. A prospective cohort study in 6 European countries of 247,249 adults, including 9,979 with COVID-19, found a higher prevalence of depression and poor sleep quality amongst individuals with COVID-19, with an increased risk of depression amongst patients who were bedridden for more than 7 days (Magnúsdóttir et al., 2022). Tackling psychological symptoms should be a priority area of focus for survivors of COVID-19 due to the increased incidence of mental health disorders when compared to patients hospitalised for other causes or similar infections such as seasonal influenza (Xie et al., 2022).

**Figure 1:**
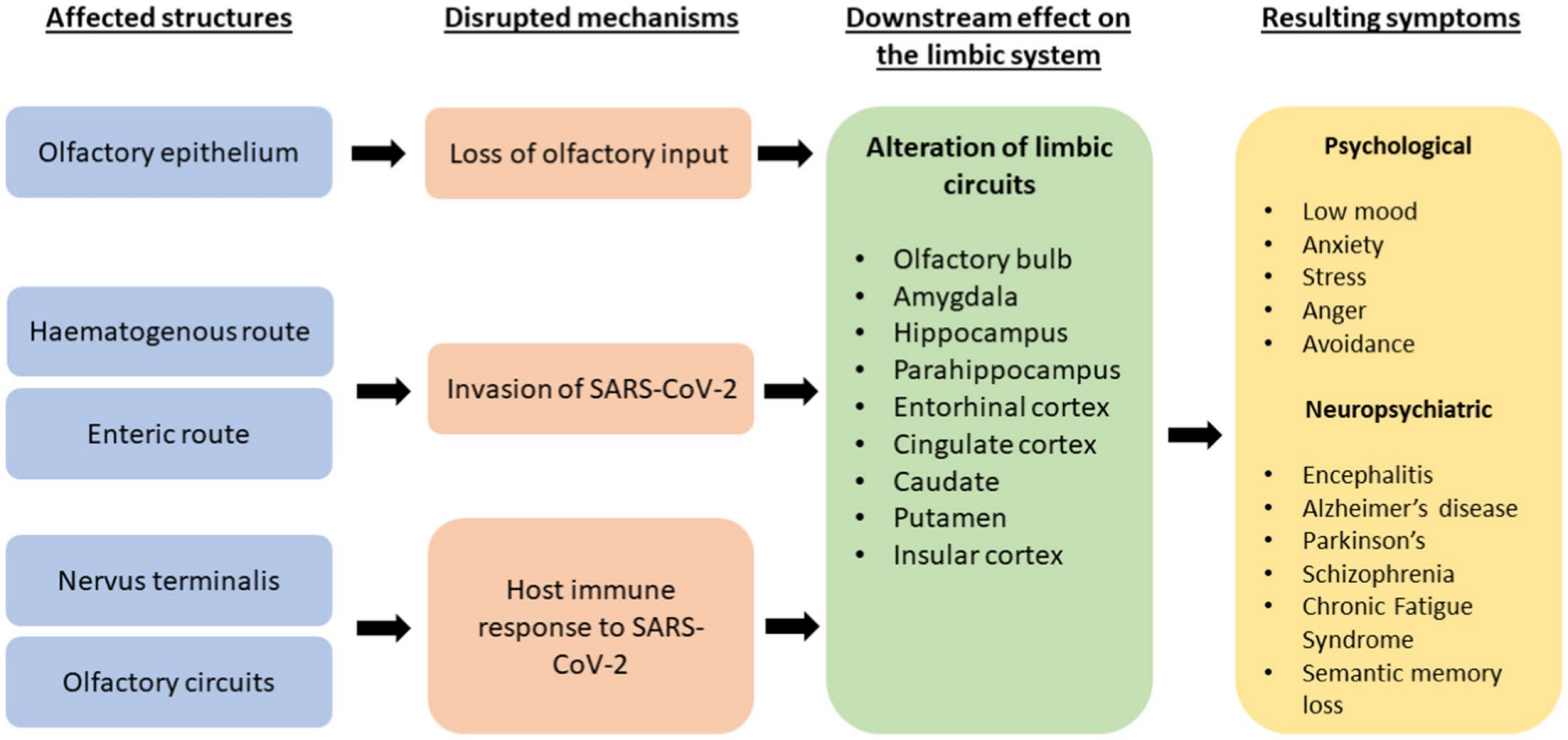
A flowchart explaining proposed mechanisms of destruction by SARS-CoV-2 and its effects on the limbic system and resultant effects on psychological, neuropsychiatric and cognitive symptoms.

Figure 1 below summarises the proposed mechanisms of destruction in acute COVID-19 infection and its downstream effects on the limbic system and its resulting cognitive, neuropsychiatric, and psychological symptoms.

## Assessment of COVID-19 olfactory dysfunction

### Clinical features of long COVID

**Figure 2:**
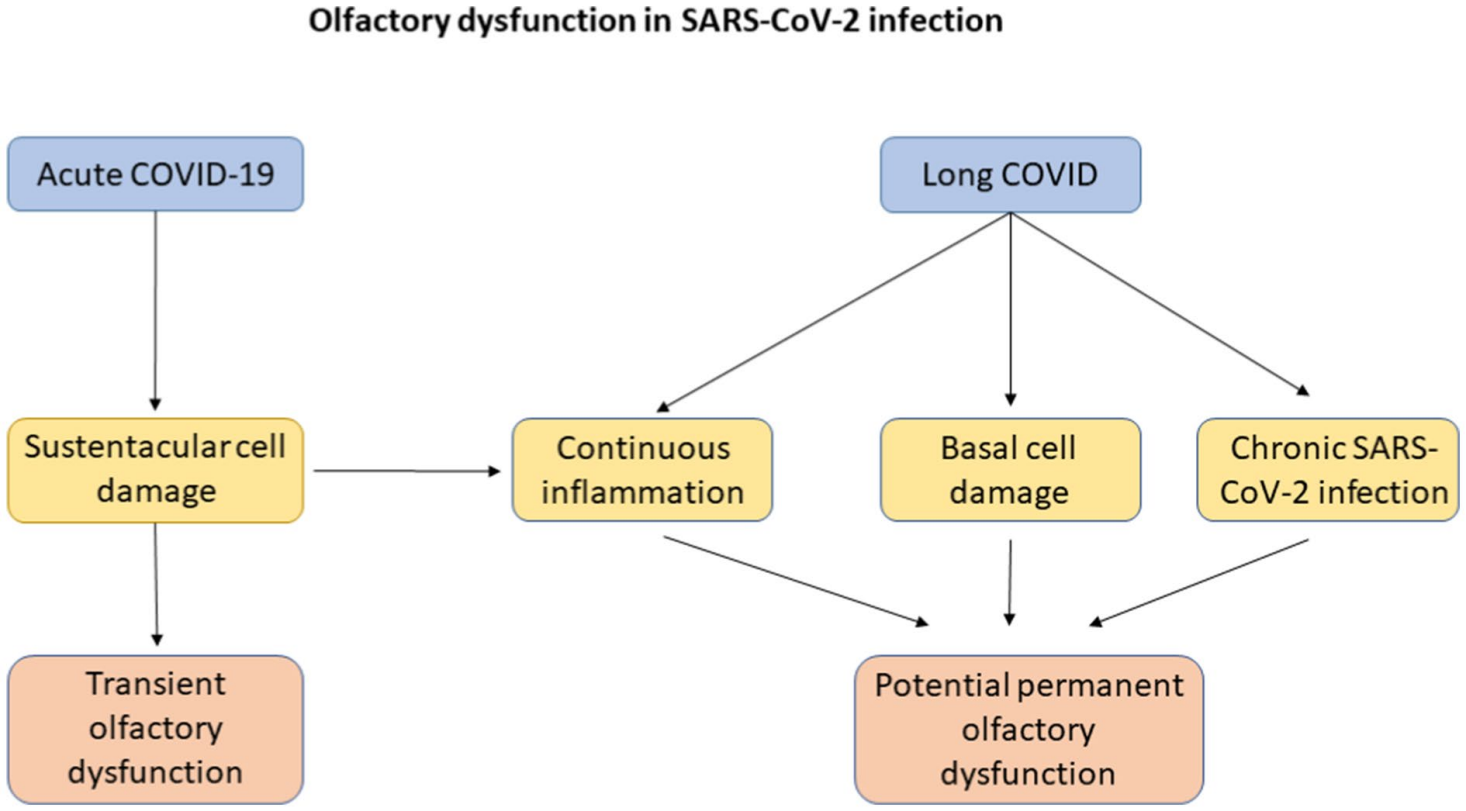
A flowchart explaining possible mechanisms of olfactory dysfunction in acute COVID-19 infection and in long COVID with its eventual progression to transient or potential permanent olfactory dysfunction.

The continuing presence of olfactory dysfunction and the potential for it to become permanent sequelae in the context of patients with long COVID presents a problem to clinicians worldwide (Gokani et al., 2022; Mendes Paranhos et al., 2022). Olfactory dysfunction in acute COVID-19 infection tends to be transient, lasting around 2–3 weeks and may be partially explained by SARS-CoV-2 having a high affinity for the sustentacular cells of the olfactory epithelium that express ACE-2 and that these cells possess substantial capacity for repair and regeneration after damage (Doty, 2021; Mendes Paranhos et al., 2022). Long term prevalence of olfactory dysfunction may be secondary to continuous inflammation, damage to basal cells and chronic SARS-CoV-2 infection in the olfactory epithelium (Liang and Wang, 2021). Chronic inflammation could result in gene expression modulation which in turn can alter the function of olfactory epithelium basal cells from neural regeneration to inflammatory signalling and immune cell proliferation (Chen et al., 2019). This has been highlighted in Figure 2.

### Olfactory dysfunction history and examination

A thorough history is required to establish the nature of olfactory dysfunction which patients are suffering from, and the extent to which their quality of life has been affected. Firstly, it is crucial to differentiate whether the patient has anosmia, hyposmia, phantosmia, or parosmia. Next, establishing a timeline of their symptoms will help identify what their smell was like before, any events that may have triggered these symptoms (besides an acute COVID-19 infection), and how they have developed over time (Luke et al., 2022).

It is important to look out for other potential causes of olfactory dysfunction. These include sinonasal disorders such as chronic rhinosinusitis, previous head trauma, surgery, or neurodegenerative disorders. Conducting a full review of all body systems will help uncover any other symptoms of long COVID. Furthermore, it is important to elicit the patient’s drug history, as many common medications are known to cause olfactory dysfunction (Schiffman, 2018). Finally, whether the patient has history of smoking, and any occupational exposure to certain hazardous chemicals is also important, as these can cause olfactory dysfunction (Schiffman, 2018; Werner and Nies, 2018).

After recording the olfactory dysfunction history, an examination of the nose is essential. Direct visualisation using fine nasal endoscopy will allow assessment of the nose, nasopharyngeal space, and olfactory cleft to rule out any causes of conductive olfactory loss (Seiden and Duncan, 2001). If the history from the patient raises suspicion of a sensorineural cause of olfactory dysfunction, a full cranial nerve examination is warranted.

### Investigating COVID-19-related olfactory dysfunction

Subjective assessments such as the Olfactory Disorders Questionnaire (ODQ) can be useful in establishing the degree of olfactory dysfunction and the impact to patients’ quality of life (Langstaff et al., 2019; Garden et al., 2023), as well as help identify qualitive symptoms such as phantosmia and parosmia.

As olfaction plays a major role in flavor perception, many patients may report a disturbance in their sense of taste. However true gustatory loss is rare and their perception of “loss” of their sense of taste is due to their olfactory dysfunction affecting retronasal olfaction (Wrobel and Leopold, 2004). Gustatory testing using Taste Strips may be a quick way in ruling out true gustatory loss. It involves using strips of filter paper consisting of four different flavours (sweet, sour, salty, bitter) in various concentrations. These strips are placed on the tongue of patients, and they are asked to identify which of the four flavours they think it is (Mueller et al., 2003).

Psychophysical olfactory testing can be performed to quantitatively measure olfactory function and confirm the presence of olfactory dysfunction (Hummel et al., 2016; Luke et al., 2022). Olfaction can be assessed orthonasally or retronasally; odours can be sniffed through the nostrils (orthonasal olfaction) or allowed to enter through the nasopharynx through the use of powders (retronasal olfaction) (Croy et al., 2014; Hummel et al., 2016; Goldberg et al., 2018). Objective testing of orthonasal olfaction can be done by using common validated psychophysical tests such as the Sniffin’ Sticks test, University of Pennsylvania Identification Test (UPSIT), the Toyota & Takagi Olfactometer, the Cross-Cultural Smell Identification Test, the Brief Smell Identification Test or the Connecticut Chemosensory Clinical Research Center (CCCRC) test (Doty et al., 1984, 1996; Cain et al., 1988; Kobal et al., 1996; Kondo et al., 1998; Menon et al., 2013; Hummel et al., 2016; Hutson et al., 2022). These tests have been established in objectively assessing the degree of olfactory dysfunction, categorizing patients into normosmia, hyposmia or anosmia (Doty et al., 1984, 1996; Cain et al., 1988; Kobal et al., 1996; Kondo et al., 1998; Menon et al., 2013; Hummel et al., 2016; Hutson et al., 2022). While the UPSIT test can be performed by the patient on their own, the Sniffin’ Stick Test requires a medical professional to administer the test. Therefore, the choice of which test to use is up to the resources and capacity of the clinic the patient is being assessed. Retronasal psychophysical testing can be performed in patients where there is a perceived mismatch between orthonasal and retronasal olfaction that is not accounted for by a gustatory component (Heilmann et al., 2002; Croy et al., 2014; Goldberg et al., 2018; Luke et al., 2022). The most common retronasal olfaction technique is the retronasal olfaction test (ROT) (Heilmann et al., 2002). This involves the placement of twenty food powders onto the tongue using squeezable plastic vials, whilst the subject’s nose is clipped. A forced-choice odour identification test method (whereby a suprathreshold odour is presented to a subject whom must identify the odour from a list of descriptors) is used with four possible options and responses recorded (Heilmann et al., 2002).

It Is important for clinicians to utilise a psychophysical test that is appropriately and culturally adapted for the subject population identified to obtain results that are reliable (Rombaux et al., 2009; Frasnelli et al., 2010; Patel et al., 2022). Furthermore, increased length of the screening testing is associated with increased reliability and validity of the results (Doty et al., 1995). Consequently, it is recommended that short screening tests be used for identification of subjects with olfactory dysfunction whereby longer tests be used to quantitatively assess the degree of olfactory dysfunction (Hummel et al., 2016; Luke et al., 2022; Patel et al., 2022).

As mentioned earlier, patients with acute COVID-19 infection and long COVID may suffer from anxiety and depression. Furthermore, patients who suffer from smell and taste disorders suffer higher rates of anxiety and depression compared to the general population. Thus, it may be beneficial in asking patients to complete validated questionnaires such as the Patient Health Questionnaire Anxiety and Depression scale to identify those who are suffering from depression and anxiety (Erskine and Philpott, 2020). A referral to the relevant mental health services can then be made.

### The role of cross-sectional imaging in investigating COVID-19-related olfactory dysfunction

The role of cross-sectional imaging in the context of COVID-19-related olfactory dysfunction has yet to be established (Whitcroft and Hummel, 2020). Computerized Tomography (CT) imaging of the paranasal sinuses and brain may be performed to exclude sinonasal or intracranial abnormalities (including malignancies) (Lund and Mackay, 1993; Higgins and Lane, 2014). Generally, structural Magnetic Resonance Imaging (MRI) has many uses in the assessment of olfactory disorders, as it allows for the assessment of the olfactory apparatus, the exclusion of asymptomatic chronic inflammation of the paranasal sinuses, the assessment of neurodegenerative disorders, the characterisation of traumatic brain injuries and the exclusion of intracranial or sinonasal neoplasms (Decker et al., 2013; Higgins and Lane, 2014; Luke et al., 2022). However, there is limited evidence to suggest a role for this modality in the context of post-infectious olfactory disorder (Hutson et al., 2022).

### Olfactory dysfunction management

Addison et al. (2021) provided an evidence-based practical guide for the management of post-infectious olfactory dysfunction, including COVID-19-related olfactory dysfunction (Addison et al., 2021). The Clinical Olfactory Working Group members emphasized the recommendation for olfactory training; a non-surgical and non-pharmacological approach to manage COVID-19-related olfactory dysfunction. Other key medical management options were discussed including steroids and vitamin A, but they highlighted the need for further research to confirm the place for the varying therapeutic options available.

### Adjunctive treatments

There are limited treatment options available for persistent COVID-19-related olfactory dysfunction as this is a relatively novel condition (Whitcroft and Hummel, 2020). However, there are numerous adjunctive management options used for post-viral olfactory dysfunction that could be used for patients who suffer from persistent olfactory dysfunction, including simple lifestyle measures, olfactory training, and traditional Chinese acupuncture (TCA).

The olfactory system is closely linked to the limbic system (Albrecht and Wiesmann, 2006). Consequently, olfactory dysfunction is associated with a deterioration in the quality of life, social skills, relationships and mental wellbeing of this patient cohort (Saniasiaya and Prepageran, 2021). Patients with olfactory dysfunction, including COVID-19-related olfactory dysfunction, report a decrease in flavour perception due to impaired retronasal olfaction (Nordin et al., 2011). This is associated with loss of or reduced appetite, as well as diminished food enjoyment (Elkholi et al., 2021; AlShakhs et al., 2022). Scheduled eating hours may address the dysregulated appetite observed (Croy et al., 2014). In addition, COVID-19-related olfactory dysfunction has been linked to depression and patient isolation (Coelho et al., 2021; AlShakhs et al., 2022). This association may be explained by the overlap between the brain areas involved in olfaction and depression, notably the orbitofrontal cortex, anterior and posterior cingulate cortices, insula, amygdala, hippocampus and thalamus (Seo et al., 2010; Marine and Boriana, 2014). Social support groups such as Fifth Sense and AbScent can play an important role in facilitating patients to emotionally accept their olfactory deficit, allowing patients to perform adaptive adjustments to their lives living with this disease (Nordin et al., 2011; Saniasiaya and Prepageran, 2021).

It is also well recognised that patients with olfactory dysfunction have concerns regarding personal safety and hygiene (Philpott and Boak, 2014). Patients with olfactory dysfunction are significantly more likely to be involved in more household accidents compared to normosmic individuals (Croy et al., 2012). Simple lifestyle measures that patients can do to keep themselves and co-habitants safe include maintaining smoke and natural gas detectors and monitoring food expiry dates and nutritional intake (Whitcroft and Hummel, 2020).

There is evidence that olfactory training (OT) is an effective and frequently used treatment option for patients with hyposmia or anosmia secondary to various aetiologies (Pekala et al., 2016; Sorokowska et al., 2017). It involves patients receiving repeated exposure to different odours over time to help improve their olfactory sensitivity (Altundag et al., 2015). Specifically, the odours typically used in OT include phenylethyl alcohol (rose scent), eucalyptol (eucalyptus scent), citronella (lemon scent) and eugenol (clove scent) (Hummel et al., 2009). Standard OT involves patients sniffing these odours (present on cotton pads within containers) twice daily for at least 20–30 s for each scent. (Kronenbuerger and Pilgramm, 2023). Hura et al. (2020) conducted a review of the treatments of post-viral olfactory dysfunction which showed OT is recommended to improve olfactory outcomes with higher concentrations, longer duration of OT and a wide variety of odours to be the most effective (Hura et al., 2020). Furthermore, OT over 4 weeks has been demonstrated to improve subjective and psychophysical testing scores in patients with persistent COVID-19-related olfactory dysfunction (De AT et al., 2022). OT is a low-cost adjunctive therapy with negligible adverse effects for patients with persistent COVID-19-related olfactory dysfunction (Whitcroft and Hummel, 2020).

TCA is a popular complementary therapy that is used for a wide variety of conditions. There have been studies investigating its use in otorhinolaryngological conditions such as allergic rhinitis, tinnitus, and sudden sensorineural hearing loss. However, there is a paucity of high-quality evidence to demonstrate its benefit (Kahn et al., 2020). There are a few studies demonstrating a possible improvement in psychophysical assessment scores with TCA in patients with post-viral olfactory loss but these have small sample sizes (Vent et al., 2010; Dai et al., 2016). To date, there is no research conducted examining the efficacy of TCA on COVID-19-related olfactory dysfunction. TCA is performed by the placement of needles in acupoints by trained professionals, with these needles kept in place for 20 min. TCA is administered 3 times a week, with each course lasting 10 sessions. There are 3–5 days of rest in between courses, and courses are repeatedly administered until the patient has received 3 months in total (Vent et al., 2010; Dai et al., 2016). Similar to OT, TCA is a cost effective, low risk complementary therapy that may benefit patients, but further research is needed to determine its efficacy in post-viral olfactory loss and COVID-19-related olfactory dysfunction.

### Pharmacological treatments

For COVID-19-related olfactory dysfunction that does not resolve spontaneously, pharmacological intervention may be indicated. A Cochrane review on intervention of persistent COVID-19-related olfactory dysfunction has highlighted the significant lack of evidence exploring the efficacy and harms of treatment for patients with COVID-19-related olfactory dysfunction (Webster et al., 2022).

### Intranasal and oral corticosteroids

Huart et al. (2021) have recently published an international expert group viewpoint that there is currently no evidence for the use of intranasal or oral corticosteroids in COVID-19-related olfactory dysfunction (Huart et al., 2021). Current literature is often underpowered and any evidence supporting the use of corticosteroids is weak (Saussez et al., 2021; Kim et al., 2022; Schepens et al., 2022). Furthermore, there is sufficient evidence that even limited systemic corticosteroid treatment can have harmful side-effects, such as increased risk of hip fractures and decompensating glaucoma (Yasir et al., 2022).

### Non-steroidal pharmacological management

#### Vitamin A

It has been theorised that vitamin A will encourage regeneration of olfactory epithelium. This is because vitamin A is converted to retinoic acid, which is thought to control olfactory progenitor cell differentiation, and can thus prevent exhaustion of stem cell supply or accumulation of non-functional immature neurones (Paschaki et al., 2013). At present, there has been no RCT that has examined the efficacy of intranasal vitamin A specifically on patients with COVID-19-related olfactory dysfunction. Promisingly, a pseudo-randomised clinical trial showed than in 124 patients with post-viral olfactory loss, a minimum clinically important difference in olfactory function was seen in 37% of those receiving intranasal vitamin A compared with 23% receiving smell training alone (Hummel et al., 2017). However, due to the unbalanced treatment groups and pseudo-randomisation, the study lacked scientific rigor that is required for further proof of concept evidence for intranasal vitamin A. There is currently an ongoing double blind randomised controlled trial (RCT), APOLLO, which aims to further explore the use of intranasal vitamin A drops in the treatment of post-viral olfactory loss (ISRCTN - ISRCTN13142505, n.d.). This in turn will provide further baseline information for this treatment option to be investigated for patients with COVID-19-related olfactory dysfunction.

#### Platelet rich plasma

Platelet rich plasma (PRP) is an autologous blood product, with supraphysiologic concentration of growth factors, and can be used in peripheral nerve regeneration. Several studies have indicated that PRP administered intravenously may be effective in improving olfactory outcomes in patients following acute COVID-19 infection (Steffens et al., 2022; Wang et al., 2022; Lechien et al., 2023); including a recent randomised controlled trial which found that patients receiving PRP injection resulted in a 3.67 increase in Sniffin’ Sticks score compared with the placebo (95% CI 0.05–7.29; *p* = 0.047) (Yan et al., 2023). However, findings were significantly underpowered with only 26 participants completing the study. Two of the studies found no adverse effects were reported, however Lechien et al. (2023) reported transient epistaxis (n = 31), parosmia related to the xylocaine spray (*n* = 10) and vasovagal episode (*n* = 2) (Lechien et al., 2023). Findings may therefore suggest that PRP could be a helpful tool in managing COVID-19-related olfactory dysfunction, however larger randomised trials are required.

#### Theophylline

Theophylline is a drug derived from methylxanthine, with it having systemic properties including smooth muscle relaxation, bronchial dilatation, and diuresis as well as having a stimulant effect on the cardiac and central nervous systems (Jilani et al., 2023). Clinically, theophylline is widely used in various obstructive respiratory pathologies including Chronic Obstructive Pulmonary Disease (COPD), asthma and infant apnoea (Jilani et al., 2023). In the context of anosmia, theophylline is suggested to improve olfactory neuroepithelium regeneration, by inhibiting phosphodiesterase and increasing secondary messengers (such as cyclic adenosine monophosphate and cyclic guanosine monophosphate) (Henkin et al., 2009, 2011). A RCT evaluating the impact of intranasal theophylline on patients with post-viral olfactory dysfunction initially indicated that there was no significant improvement in smell between the theophylline group compared with the placebo saline irrigation (Lee et al., 2022). However, authors hypothesized that the dosage of theophylline could be safely elevated, and thus conducted a phase 2 trial specifically on patients with COVID-19-related olfactory dysfunction (Gupta et al., 2022). At the higher dose, mixed model analysis revealed that the change in UPSIT score was not significantly different between the two groups. These findings were limited by the small sample size of 45 participants and the use of subjective assessments of olfactory dysfunction. Larger studies, using more objective testing methods, are warranted to further investigate the impact and efficacy of intranasal theophylline on patients with COVID-19-related olfactory dysfunction.

#### Ultramicronized palmitoylethanolamide and luteolin supplements

Ultramicronized palmitoylethanolamide and luteolin (PEA-LUT) are anti-inflammatory and neuroprotective agents. One hypothesis is that COVID-19-related olfactory dysfunction may be due to neuroinflammatory results within the olfactory bulb and central nervous system, therefore PEA-LUT may have a potential role in its management. A RCT of 185 patients with COVID-19-related olfactory dysfunction found that those treated with PEA-LUT oral supplements plus olfactory training showed significantly greater improvement in Sniffin’ Sticks score compared with controls (Di Stadio et al., 2022). By the 90-day endpoint, there was greater than a ten-fold prevalence of anosmia in the control versus intervention. Although providing promising results, further longitudinal studies are required for clarifying optimal timing and dosing parameters of PEA-LUT for patients with limited or absent recovery from COVID-19-related olfactory dysfunction and to also evaluate the effect of PEA-LUT on neuroinflammation by measuring specific neuroinflammatory biomarkers.

#### Zinc sulphate

Zinc is an important trace metal in the human body, and it regulates the differentiation, proliferation, maturation and function of lymphocytes and other leucocytes (Jeong and Eide, 2013; Gammoh and Rink, 2017; Abdelmaksoud et al., 2021). Consequently, it was hypothesised that zinc deficiency may contribute to COVID-19-related olfactory dysfunction due to these patients being more susceptible to severe acute COVID-19 infection and its associated complications. However, a recent study has found that serum zinc levels in patients with acute COVID-19 infection were not significantly different between those with the presence of or those with the absence of olfactory and/or gustatory dysfunction (Abdelmaksoud et al., 2021). Interestingly, they did find that the median duration of olfactory and/or gustatory dysfunction was significantly shorter in patients who received oral zinc supplements. Further longitudinal studies should be conducted to investigate the impact and efficacy of oral zinc supplements in the role of treating COVID-19-related olfactory dysfunction.

#### Buffer solutions

Free calcium plays a fundamental role in peripheral olfactory processing, including feedback inhibition. Thus, it is proposed that the reduction of intranasal free calcium with buffer solutions may improve olfactory function in patients with olfactory impairment (Whitcroft and Hummel, 2019). Examples of buffer solutions include sodium citrate, tetra sodium pyrophosphate (TSPP) and sodium gluconate, which are discussed below.

##### Sodium citrate

Sodium citrate administered intranasally can modulate the cascade of olfactory receptor transduction (Whitcroft and Hummel, 2019). At present there is currently no RCT investigating the efficacy of intranasal sodium citrate in patients with COVID-19-related olfactory dysfunction. However, an improvement in olfactory threshold was seen in a prospective placebo-controlled trial, whereby intranasal sodium citrate was trialled against intranasal sodium chloride treatment for 57 patients with olfactory loss (Whitcroft et al., 2016). Furthermore, in a prospective placebo-controlled trial with 49 patients exclusively with post-viral olfactory dysfunction, intranasal sodium citrate showed significant improvement in the compound threshold and ident cation scores but nil change in odour or threshold identification when compared to the placebo (Whitcroft et al., 2017). A single application of 0.5 mL of sodium citrate per nostril, compared to sterile water in a RCT of 55 patients with non-conductive olfactory dysfunction, was shown to have statistically significant improvement in olfactory function using olfactory thresholds for phenyl ethyl alcohol, 1-butanol, and eucalyptol, with thresholds measured up to 2 h post intervention (Philpott et al., 2017). It is proposed that the sodium citrate solution administered nasally binds to the free calcium ions in the nasal mucus, thus reducing the free calcium available in the nasal mucosa (Philpott et al., 2017). All the aforementioned studies lack robust long-term data as well as data specific to patients with COVID-19-related olfactory dysfunction, and this will need to be addressed in future RCTs in order to explore the clinical applications and efficacy of sodium citrate as a buffer solution in this patient cohort.

##### Tetra sodium pyrophosphate

Tetra sodium pyrophosphate (TSPP) is a calcium chelating agent that lowers calcium concentration (Shirashoji et al., 2016). Reduced calcium has been suggested to increase sensitivity to odorants, as shown utilising sodium citrate to improve olfactory function (Philpott et al., 2017). A randomised controlled trial, on 64 patients with COVID-19-related olfactory dysfunction, claimed to find that there was a statistically significant improvement in Sniffin’ Stick scores between those treated with intranasal TSPP compared with sodium chloride, but the study was underpowered for the minimum clinically important difference in the Sniffin’ Sticks (Abdelazim et al., 2022). This may be due to the role of intranasal TSPP as a chelating agent, as the TSPP group had a statistically significant lower nasal calcium concentration than those treated with sodium chloride. Larger, higher-powered studies will be required to further investigate the role of intranasal TSPP as a buffer solution in treating COVID-19-related olfactory dysfunction.

##### Sodium gluconate

Similarly, to TSPP, sodium gluconate has also shown to be a highly efficient chelating agent (Fiume et al., 2019). It has also shown some potential in the use to treat COVID-19-related olfactory dysfunction, with a statistically significant improvement in Sniffin’ Stick scores in those receiving intranasal sodium gluconate at 1 month (Abdelazim and Abdelazim, 2022). As with TSPP, these studies are underpowered at best, and will require larger, well powered studies to investigate its efficacy as a buffer solution in improving olfactory function in this patient cohort.

### Proposed algorithm for investigating COVID-19-related olfactory dysfunction

**Figure 3:**
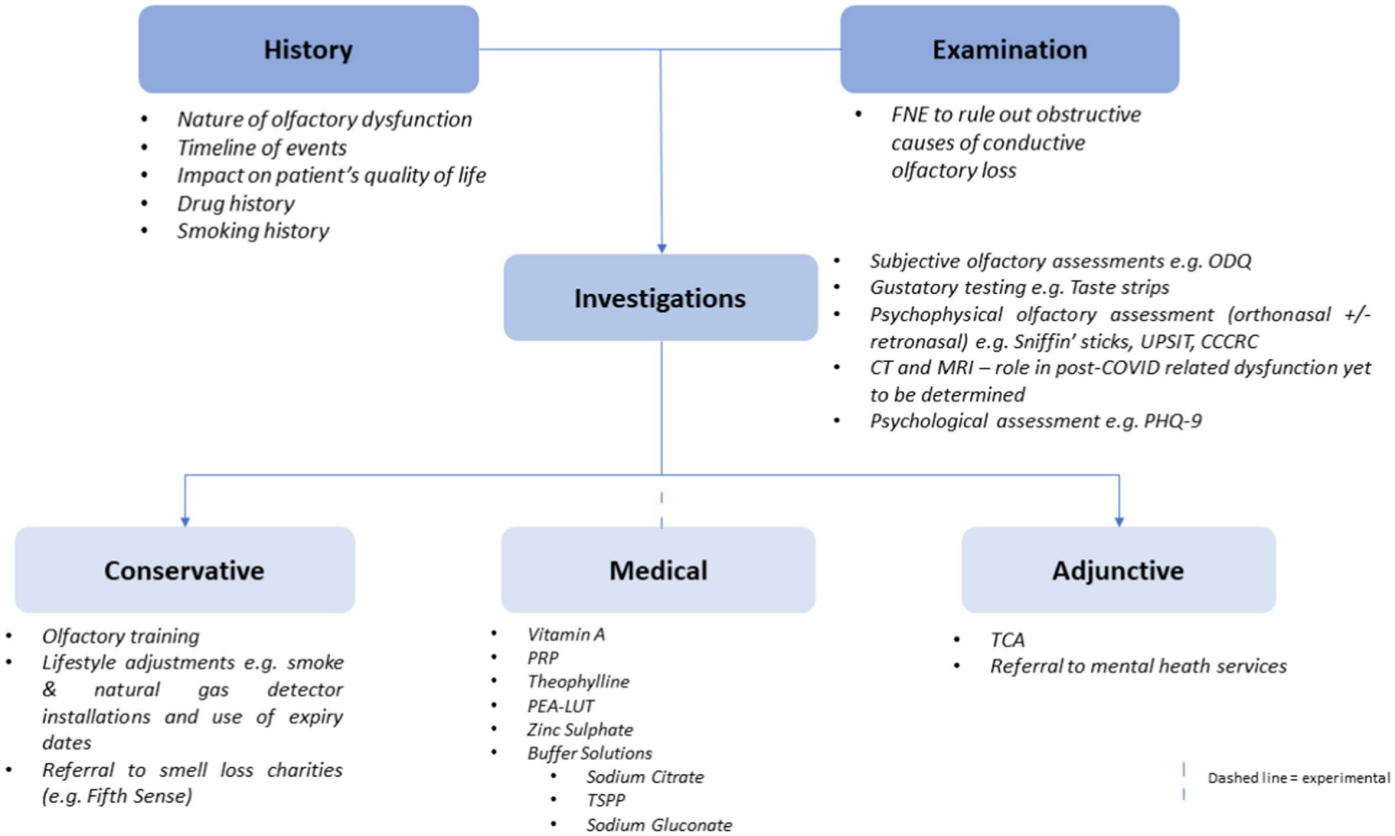
A flowchart illustrating a proposed algorithm for clinicians to follow when managing a patient with suspected COVID-19-related olfactory dysfunction.

Utilising the aforementioned evidence, the authors propose an algorithm for clinicians to utilise when presented with patients with possible COVID-19-related olfactory dysfunction. This can be seen in Figure 3 below.

In summary, the efficacy of the medical management of COVID-19-related olfactory dysfunction remains experimental at best, with studies for the different treatment strategies either being underpowered or not performed on patients with COVID-19-related olfactory dysfunction. Future larger, highly powered studies which utilises validated olfactory assessment scores will provide light on the efficacy of these treatments.

## Conclusion

Acute COVID-19 infection and long COVID have strong associations with psychological, neuropsychiatric, and cognitive symptoms. A systematic and holistic approach with history taking and examination particularly with nasal endoscopy can determine the impact that COVID-19-related olfactory dysfunction has on the patient. Specific olfactory disorder questionnaires can demonstrate the impact on quality of life, while psychophysical testing can objectively assess and monitor olfaction over time. The role of cross-sectional imaging is not yet described for COVID-19-related olfactory dysfunction. Management options are limited to conservative adjunctive measures, with medical therapies having a yet unproven role in the treatment of this disorder. Further research, in the form of larger, highly powered RCTs will be needed to examine the efficacy of pharmacological and non-pharmacological interventions for patients with COVID-19-related olfactory dysfunction.

## Author contributions

LJ conceived the idea and critically revised the article. All authors satisfied the ICMJE criteria for authorship. CP was the lead supervisor for the project.

## Conflict of interest

CP reports grants from NIHR, ESPRC, and ENT UK, personal fees from Stryker, Abbott, and Olympus, outside the submitted work, and Trustee of Fifth Sense.

The remaining authors declare that the research was conducted in the absence of any commercial or financial relationships that could be construed as a potential conflict of interest.

## Publisher’s note

All claims expressed in this article are solely those of the authors and do not necessarily represent those of their affiliated organizations, or those of the publisher, the editors and the reviewers. Any product that may be evaluated in this article, or claim that may be made by its manufacturer, is not guaranteed or endorsed by the publisher.
